# Supplementary material for: Climate variation and serotype competition drive dengue outbreak dynamics in Singapore
Source: Nat Commun. 2025 Nov 22;16:11364. doi: 10.1038/s41467-025-66411-6 (PMC12727700; doi:10.1038/s41467-025-66411-6)
Supplement: Supplementary file 1 — Supplementary Information [file 41467_2025_66411_MOESM1_ESM.pdf]

Supplementary information for:

Climate variation and serotype competition drive dengue outbreak dynamics in Singapore

Emilie Finch[1,2], Chia-Chen Chang[3,4], Adam Kucharski[1], Shuzhen Sim[3], Lee-Ching Ng[3,5,6], Rachel Lowe[1,6,7,8,9]

[1]Centre of Mathematical Modelling of Infectious Diseases, London School of Hygiene and Tropical Medicine, London, UK

[2]Department of Genetics, University of Cambridge, UK

[3]National Environment Agency, Singapore

[4]Department of Biological Sciences, National University of Singapore, Singapore

[5]School of Biological Sciences, Nanyang Technological University, Singapore

[6]Saw Swee Hock School of Public Health, National University of Singapore, Singapore

[7]Centre on Climate Change and Planetary Health, London School of Hygiene and Tropical Medicine, London, UK

[8]Barcelona Supercomputing Center (BSC), Barcelona, Spain

[9]Catalan Institution for Research and Advanced Studies (ICREA), Barcelona, Spain

*Corresponding authors: Emilie Finch (ef507@cam.ac.uk) and Rachel Lowe (rachel.lowe@bsc.es)

Supplementary Note

Model priors and hyperparameter formulations

The weekly random effect $\delta_{w[t]}$ was assigned a random walk 2 prior distribution (second order difference prior distribution) where we assume that second order increments follow a Gaussian distribution with zero mean and precision τ. This was defined to be cyclic (so the first week of the year is dependent on the last two weeks of the previous year). We included independent and identically distributed (iid) random effects for each year $\gamma_{a[t]}$. Non-linear covariates were modelled by setting a random walk 2 prior on the coefficients of the covariates. We used penalized complexity priors (PC priors) for precision τ, with hyperparameters $\sigma_0 = 0.5$ and $\alpha = 0.01$ for all non-linear covariates and random effects in the model. The PC prior is defined on the standard deviation $\sigma = \tau^{-1/2}$ such that $P(\sigma > \sigma_0) = \alpha$. these penalize departure from $\sigma = 0$. PC priors follow the principle of parsimony, favouring a base model (where $\sigma = 0$) unless evidence is provided against it (1).

Table 1: Covariates tested in model selection

Variable name	Covariate class	Variable type
Minimum temperature °C	Temperature	Numeric
Mean temperature °C	Temperature	Numeric
Maximum temperature °C	Temperature	Numeric
Absolute humidity g/m^3	Humidity	Numeric
Relative humidity %	Humidity	Numeric
Total precipitation (mm)	Rainfall	Numeric
Number of days without rain	Rainfall	Numeric or categorical
Number of days with heavy rain	Rainfall	Numeric or categorical
Number of days with moderate or heavy rain	Rainfall	Numeric or categorical
Number of days with consecutive rainfall	Rainfall	Numeric or categorical
Niño 3.4 sea surface temperature anomalies (SSTA)	ENSO	Numeric
Serotype proportions	Serotype	Numeric
Serotype growth rates	Serotype	Numeric
Dominant serotype	Serotype	Factor
Switch in dominant serotype	Serotype	Binary
Time since switch in dominant serotype	Serotype	Numeric

Table 2: Details of model selection

We conducted forward stepwise selection, grouping climatic indicators into classes of covariate including: temperature, rainfall, humidity and Niño 3.4. At each step of model selection, the best performing variable was carried forwards and tested against all variables in remaining climate classes. Note that at the third stage of model selection the 12 week total days without rain performed similarly well to 12 week total precipitation (mm); we selected the former due to the high influence of outlier precipitation values on the estimated effect size. Finally, we did not include absolute humidity as there was evidence for no effect on dengue incidence and only marginal improvements in model adequacy criteria.

Step	Variable	WAIC	DIC	Rsq
1	Maximum temperature (12 week running average, non-linear, no lag)	12830.96	12829.96	0.067
2	Maximum temperature (12 week rolling average, no lag) + Niño 3.4 (12 week rolling average, non-linear, 1 month lag)	12786.84	12785.10	0.103
3	Maximum temperature (12 week rolling average, no lag) + Niño 3.4 (12 week rolling average, non-linear, 1 month lag) + 12 week total days without rain (no lag)	12756.24	12754.75	0.139
4	Maximum temperature (12 week rolling average, non-linear, no lag) + Niño 3.4 (12 week rolling average, non-linear, 1 month lag) + 12 week total days without rain (non-linear, no lag) + absolute humidity (4 month lag)	12748.57	12746.83	0.146
Adding serotype	Maximum temperature (12 week rolling average, non-linear, no lag) + Niño 3.4 (12 week rolling average, non-linear, 1 month lag) + 12 week total days without rain (non-linear, no lag) + time since switch in dominant serotype (non-linear)	12498.13	12498.27	0.331

Table 3: Details of selected models

Full model formulae for full *climate and serotype* model and other models compared in the main text.

Final models	Formula
Climate and serotype	Time since switch in dominant serotype (non-linear) + maximum temperature °C (12 week average, non-linear) + days without rain (12 week total, non-linear) + Niño 3.4 SSTA (12 week average with a 4 week lag) + $\gamma_{a[t]} + \delta_{w[t]}$
Climate only	Maximum temperature °C (12 week average, non-linear) + days without rain (12 week total, non-linear) + Niño 3.4 SSTA (12 week average with a 4 week lag) + $\gamma_{a[t]} + \delta_{w[t]}$
Serotype only	Time since switch in dominant serotype (non-linear) + $\gamma_{a[t]} + \delta_{w[t]}$
Seasonal baseline	$\delta_{w[t]}$

Table 4: Forecast metrics over different forecast horizons

Forecast skill metrics for each candidate model at forecast horizons from 0-8 weeks. Metrics include: CRPS (lower scores are better) CRPSS (higher scores are better), Brier score (lower scores are better), AUC (higher scores are better), false alarm rate (lower scores are better) and hit rate (higher scores are better). The trigger threshold which maximises the AUC for each model is also shown. The best score for each forecast metric and horizon is shown in bold.

Horizon	Model	CRPS	CRPSS	Brier score	AUC	False alarm (%)	Hit rate (%)	Trigger threshold (%)
0	*Climate and serotype*	**50**	**59.5**	**0.0544**	**98.4 (95% CI: 97.69-99.03)**	**2.08**	**91.6**	71.4
0	*Serotype only*	63.2	48.9	0.062	97.8 (95% CI: 97-98.65)	2.54	90.7	65.4
0	*Climate only*	57.5	53.5	0.0655	97.8 (95% CI: 96.95-98.6)	3.92	93.4	54.7
0	*Seasonal baseline*	124	0	0.232	73.4 (95% CI: 69.8-77.09)	11.8	65.2	37.8
2	*Climate and serotype*	**56.4**	**54.9**	**0.0623**	**97.8 (95% CI: 96.96-98.58)**	3.46	91	64
2	*Serotype only*	70	43.9	0.0713	97 (95% CI: 95.99-98.03)	**2.77**	88.9	65.6
2	*Climate only*	62.3	50.1	0.0722	97 (95% CI: 96.05-98.03)	4.38	**92.5**	49.6
2	*Seasonal baseline*	125	0	0.235	71 (95% CI: 67.22-74.79)	14.6	66.7	37
4	*Climate and serotype*	**67.9**	**46.3**	**0.0773**	**96.5 (95% CI: 95.44-97.62)**	5.54	**90.4**	49.2
4	*Serotype only*	82.4	34.9	0.0894	94.9 (95% CI: 93.45-96.4)	5.19	87.1	50.6
4	*Climate only*	71.7	43.4	0.0863	95.4 (95% CI: 94.11-96.78)	**5.07**	89.5	49.2
4	*Seasonal baseline*	127	0	0.24	68 (95% CI: 64.11-71.94)	15.6	64.3	37
6	*Climate and serotype*	86.5	32.4	**0.0889**	**95.4 (95% CI: 94.1-96.74)**	6.23	**89.5**	45.4
6	*Serotype only*	104	18.8	0.105	93.1 (95% CI: 91.31-94.86)	5.77	83.2	54.6

6	*Climate only*	**86.2**	**32.7**	0.0982	94 (95% CI: 92.37-95.57)	**4.38**	83.5	52.5
6	*Seasonal baseline*	128	0	0.243	65.3 (95% CI: 61.35-69.35)	17.1	64.6	36.2
8	*Climate and serotype*	86.5	32.4	**0.098**	**94.2 (95% CI: 92.65-95.75)**	6.92	**90.7**	41.7
8	*Serotype only*	104	18.8	0.123	90.9 (95% CI: 88.79-92.98)	7.38	81.4	51.5
8	*Climate only*	**86.2**	**32.7**	0.115	92.1 (95% CI: 90.24-93.92)	**6**	80.5	51.6
8	*Seasonal baseline*	128	0	0.245	64.3 (95% CI: 60.27-68.33)	14.2	56.5	37.8

CRPS: continuous ranked probability score; CRPSS: continuous ranked probability skill score; AUC: area under the curve; CI: confidence interval

Table 5: Variables used for prediction for each forecast horizon

Forecast horizon	Temperature	Precipitation	ENSO	Serotype
0	12 week average maximum temperature °C, 0 week lag	12 week total days with no rain, 0 week lag	12 week rolling average Niño 3.4 SSTA	Time since serotype switch, 0 week lag
2	10 week average maximum temperature °C	10 week total days with no rain scaled up by 1.2	12 week rolling average Niño 3.4 SSTA	Time since serotype switch + 2
4	8 week average maximum temperature °C	8 week total days with no rain,scaled up by 1.5	12 week rolling average Niño 3.4 SSTA	Time since serotype switch + 4
6	6 week average maximum temperature °C	6 week total days with no rain, scaled up by 2	10 week rolling average Niño 3.4 SSTA	Time since serotype switch + 6
8	4 week average maximum temperature °C	4 week total days with no rain, scaled up by 3	8 week rolling average Niño 3.4 SSTA	Time since serotype switch + 8

Figure 1: Dengue cases and outbreak threshold

Figure showing weekly reported dengue cases from 1 January 2000 – 31 December 2022 (grey bars) and seasonal moving 75th percentile outbreak threshold (green line) and the endemic channel threshold used operationally in Singapore (brown line). For a given month and year, we defined a seasonal moving 75th percentile outbreak threshold using the 75th percentile of weekly cases in that month using all years up to, but not including, the current year. Here, the endemic channel threshold is calculated as two standard deviations in excess of mean cases over the past 5 years, with outliers removed. Outliers are defined as any weekly cases greater than the threshold for that year.

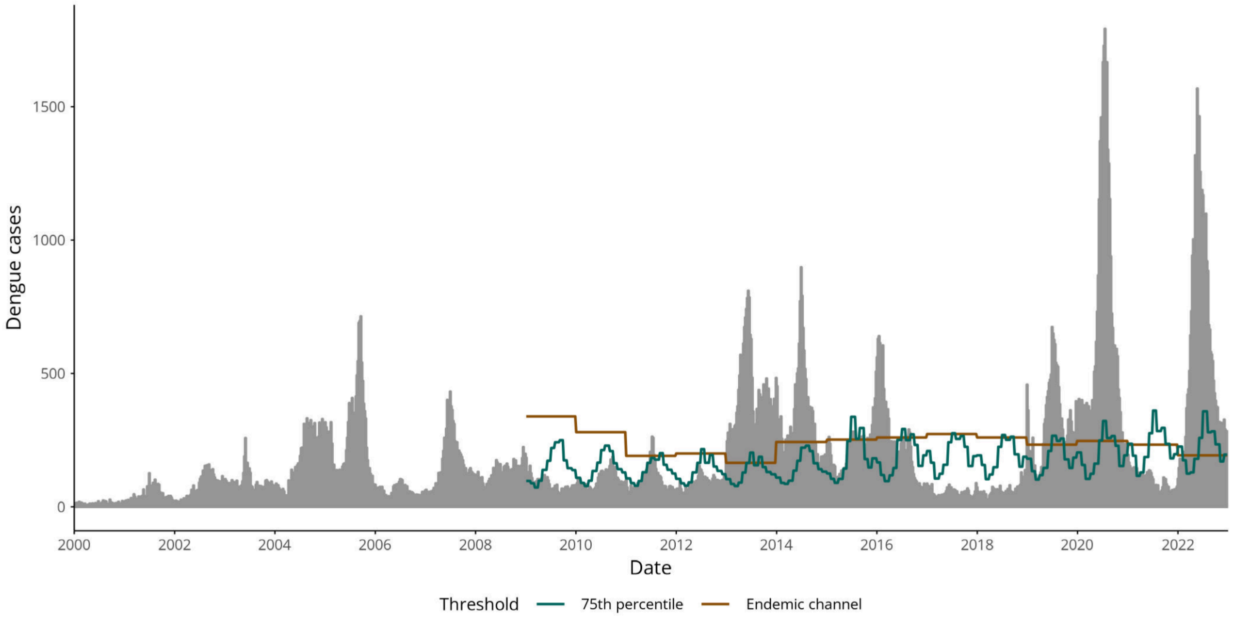

Figure 2: Schematic of time series cross-validation design to generate forecasts

Schematic showing how *h* week ahead forecasts are generated using time series cross-validation. Blue blocks represent the training data, orange blocks time points to be forecasted and grey points represent data not included when generating forecasts. Columns show the training/testing design for 2-step ahead and 3-step ahead forecasts respectively, while rows show different example municipalities. We use an expanding window approach where, for each forecast horizon *h*, we train the model on data available until week *t* - *h*. We then predict dengue incidence in week *t,* using lagged covariates. When conducting model evaluation for week t and forecast horizon h, we used the same expanding window time series cross-validation approach (Methods). We trained the model using the final selected covariates on data available until week $t - h$. We then predicted dengue incidence in week t using the best approximation of each climatic covariate. For example, considering only the temperature covariate for simplicity, when predicting dengue cases with a 4 week ahead time horizon, we fit the model up until week $t - h$ using a 12 week running average temperature to estimate model parameters. Then, using these estimated model parameters, we predict dengue cases at week t by inputting 8 week running average temperature with a 4 week lag (alongside other lagged covariates). This allows us to preserve the key relationships between climate and serotype covariates, and dengue cases that we estimate in full model fitting and then use the best climate data available at different lead times to generate forecasts for early warning.

Note that this differs from the design used for model evaluation (shown in Figure 3 of the main text), where data until $t - 1$ is used to train the model. Climatic data up to time t is then used to generate predictions for dengue incidence at time t. Serotype covariates are constructed using data until $t - 1$ as serotype frequencies are dependent on case counts.

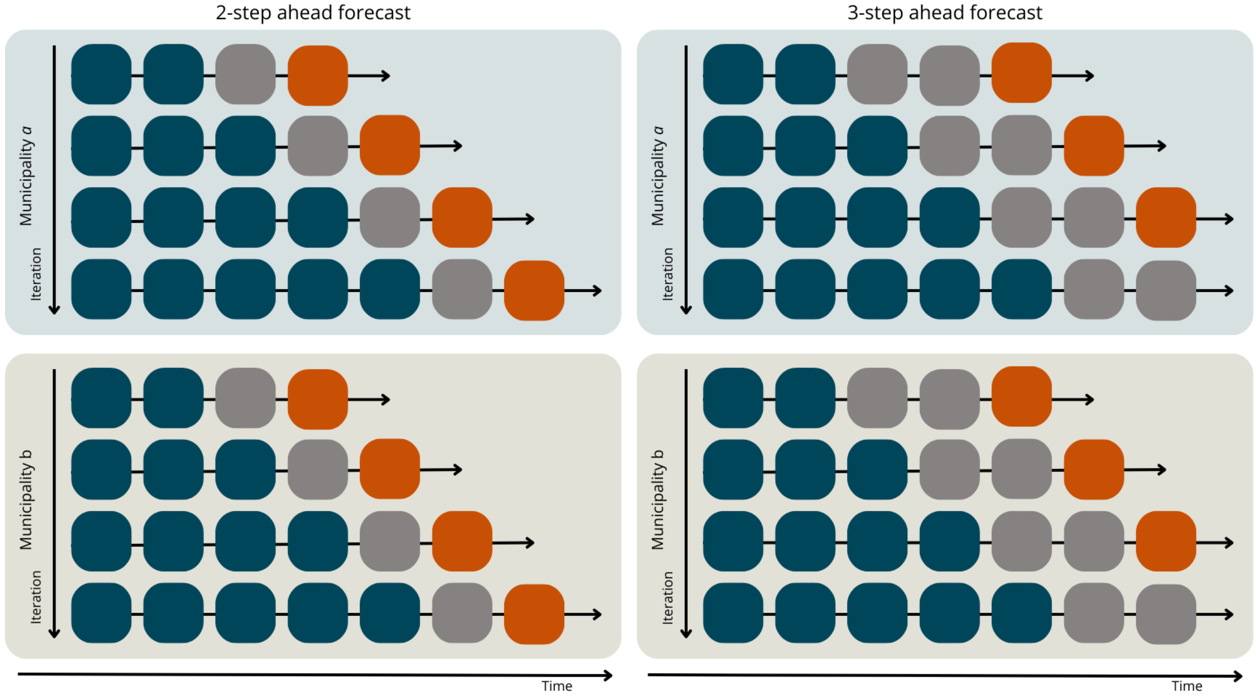

Figure 3: Dengue forecasts for early warning at 2-8 week forecast horizons

Figure showing time series of cross-validated posterior predictions of dengue cases for each model from 2009 – 2022 at 2 - 8 week forecast horizons. Columns for 4 weeks ahead and 8 weeks ahead are also shown in Figure 5 of the main text. Coloured lines show the median posterior prediction of weekly dengue cases, shaded areas show the 95% prediction interval and the dark grey line shows the data. From top to bottom the figure shows: predictions for the final selected *climate and serotype* model with weekly and yearly random effects $\gamma_{a[t]} + \delta_{w[t]}$ in purple; predictions for a *climate only* model with random effects in pink; predictions for a *serotype only* model with random effects in green; and predictions from a *seasonal baseline* model with only weekly random effects $\delta_{w[t]}$ in orange. From left to right each column shows forecasts at 2, 4, 6 and 8 weeks ahead respectively.

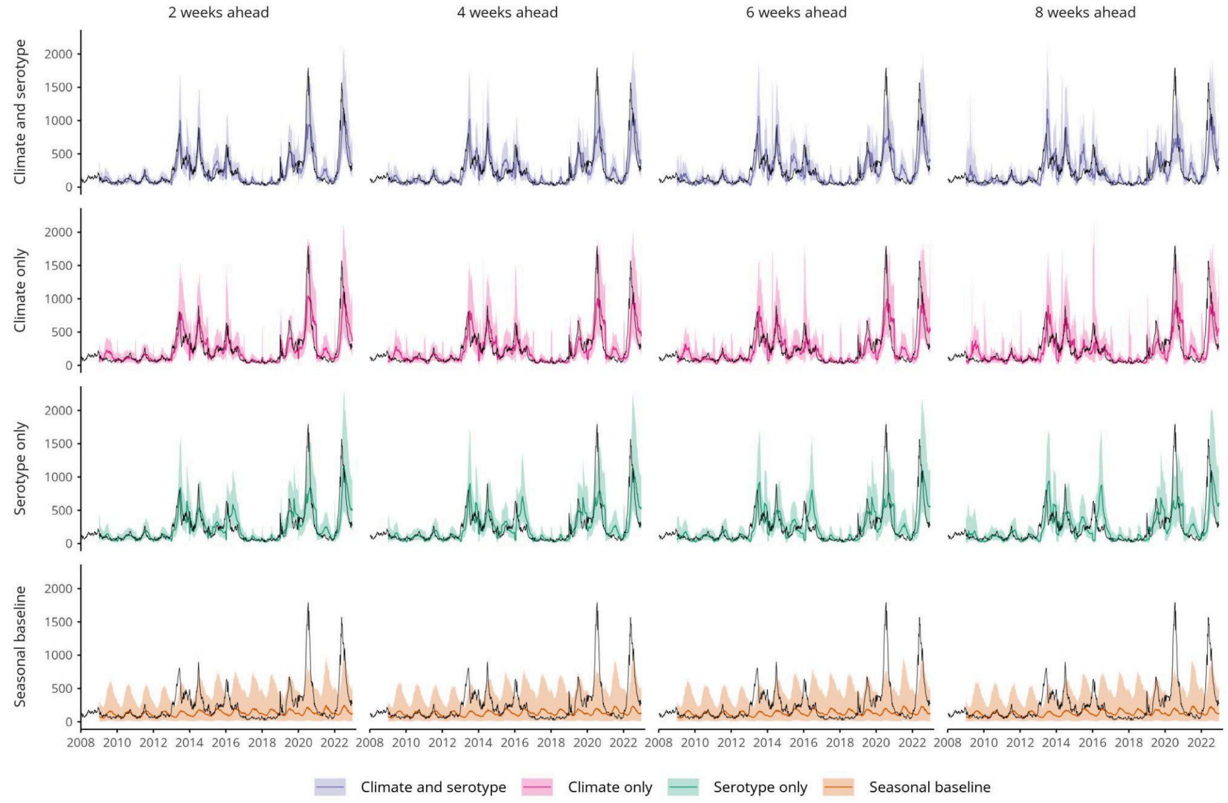

Figure 4: Predictive performance by month for different forecast horizons and covariate models

Figure showing the continuous ranked probability score on a log scale for each model by month of the year, faceted by forecast horizon. Here we can compare the performance of the model early in the dengue season (from January to May when dengue cases typically start to rise), during peak months (from June - October) and late in the season (from October - December). As CRPS values typically scale with the magnitude of the forecast target (and the magnitude of dengue cases varies substantially by month in the dengue season), here, we calculate the CRPS of log-transformed forecasts and case counts. This generates CRPS values that are independent of the magnitude of the predicted case count, enabling an evaluation of predictive performance by month (2). The CRPS can take values between 0 and infinity, with smaller values indicating better performance. For our covariate models (*climate and serotype, climate only and serotype only)* we found highest CRPS (log scale) values in January across all forecast horizons (corresponding to worst predictive performance). By February performance was generally comparable with performance throughout the rest of the season. The *climate and serotype* and *serotype only* models show a slight dip in predictive performance in July (typically aligning with the peak of the dengue season). Contrastingly, the *seasonal baseline* model shows worse predictive performance during the peak of the dengue season with best predictive performance late in the dengue season.

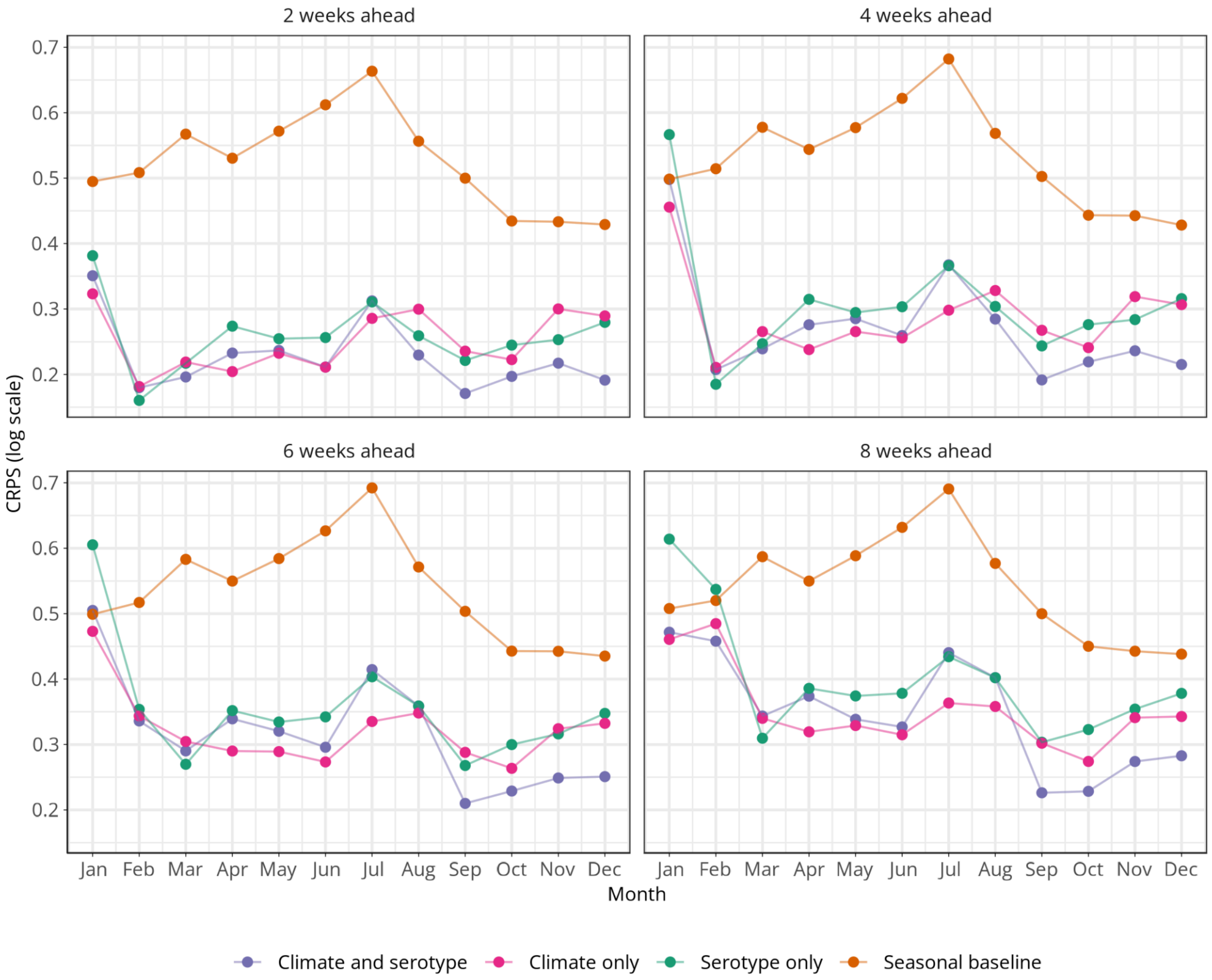

Figure 5: Dengue forecasts for early warning at 2-8 week forecast horizons showing covariate models without yearly random effects

Figure showing time series of cross-validated posterior predictions of dengue cases for each model from 2009 – 2022 at 2 - 8 week forecast horizons. Here, unlike Figure 5 in the main text, the *climate and serotype*, *climate only* and *serotype only* models do not include a yearly random effect $\gamma_{a[t]}$. Coloured lines show the median posterior prediction of weekly dengue cases, shaded areas show the 95% prediction interval and the dark grey line shows the data. From top to bottom the figure shows: predictions for the final selected *climate and serotype* model with weekly random effects $\delta_{w[t]}$ in purple; predictions for a *climate only* model with weekly random effects in pink; predictions for a *serotype only* model with weekly random effects in green; and predictions from a *seasonal baseline* model with only weekly random effects in orange. From left to right each column shows forecasts at 2, 4, 6 and 8 weeks ahead respectively.

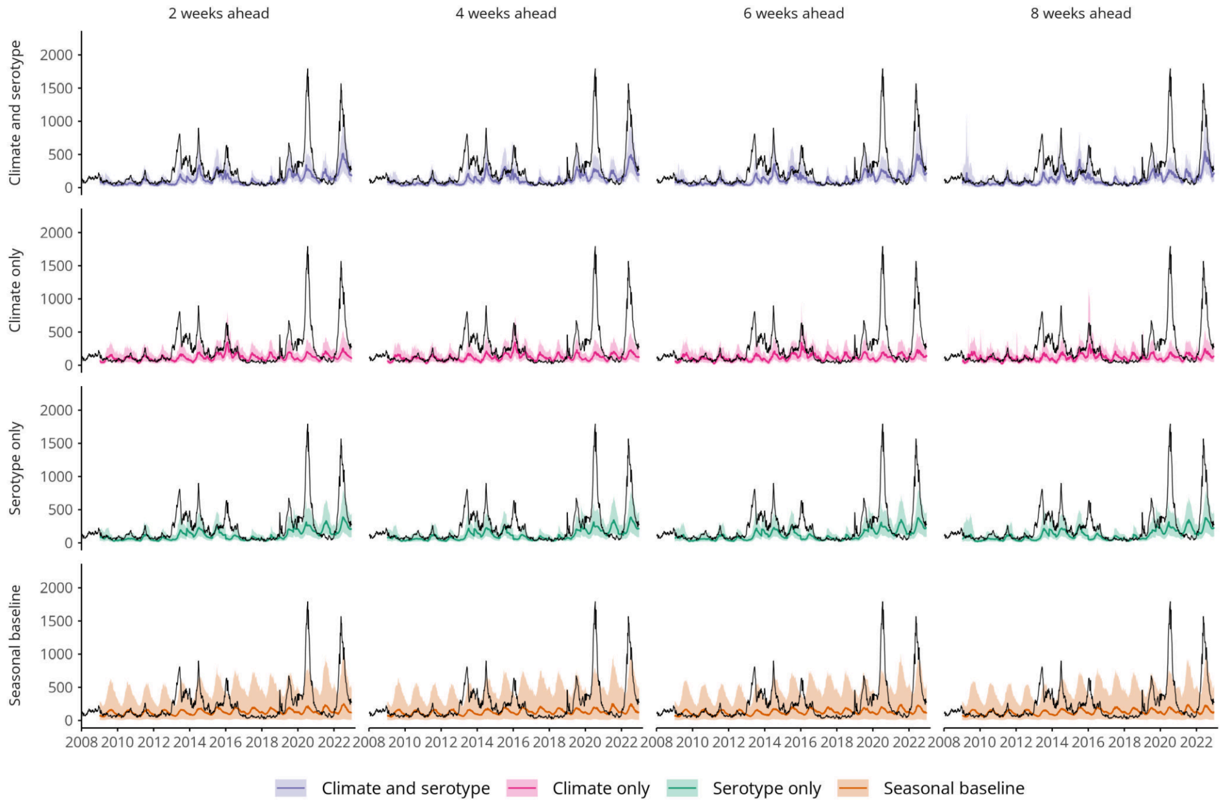

Figure 6: Predictive performance over different forecast horizons for covariate models without yearly random effects

Figure showing forecast metrics for each model across all forecast horizons from 2009 – 2022. Unlike Figure 6 in the main text, here covariate models do not include a yearly random effect $\gamma_{a[t]}$. From top left to bottom right these show: interval coverage %, bias; CRPS (continuous ranked probability score), CRPSS (continuous ranked probability skill score, %), Brier score, AUC (area under the curve, %), hit rate (%) and false alarm rate (%). Interval coverage shows the percentage of observations falling inside a given prediction interval. A perfectly calibrated forecast would have coverage equal to the nominal prediction interval; that is, 95% coverage equal to 95% and 50% coverage equal to 50%, indicated by dashed horizontal lines. Bias measures the relative tendency of the model to over- or under-predict, and is bounded between -1 and 1, with 0 indicating unbiased forecasts. The CRPS can take values between 0 and infinity, with smaller values indicating better performance. The CRPSS indicates the relative improvement of each covariate model over the *seasonal baseline* model and can take values from 0%, indicating that the model performs the same as the baseline, and 100%, indicating perfect forecasting skill. The Brier score can take values from 0 – 1, with smaller values indicating better performance. The AUC can take values from 0 – 100% with 100% indicating perfect classification. Hit rate and false alarm rate also take values from 0 – 100% with higher and lower values indicating better performance respectively.

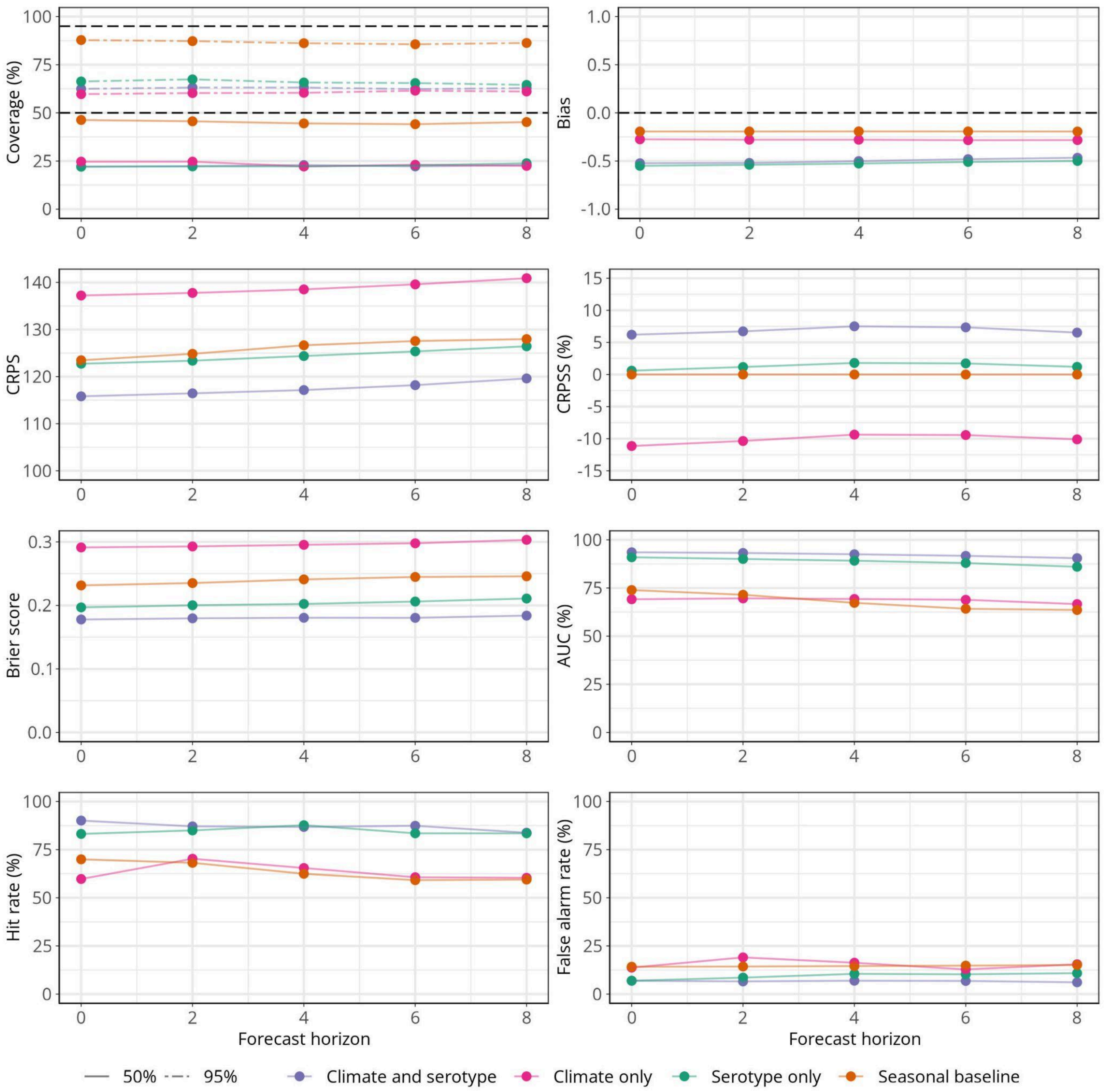
Coverage (%)
Bias
CRPS
CRPSS (%)
Brier score
AUC (%)
Hit rate (%)
False alarm rate (%)
Forecast horizon
Forecast horizon
50%
95%
Climate and serotype
Climate only
Serotype only
Seasonal baseline

Figure 7: Comparing proposed INLA model dengue forecasts with the National Environment Agency's LASSO model forecasts

Figure comparing case forecasts from the proposed *climate and serotype* INLA model with an updated version of a LASSO model developed by the National Environment Agency of Singapore (NEA) for the 2019, 2020, 2021 and 2022 dengue seasons at 2-8 week forecast horizons (3). Coloured lines show the median posterior prediction of weekly dengue cases, shaded areas show the 95% prediction interval and the dark grey line shows the data. From left to right the figure shows: predictions for the proposed *climate and serotype* INLA model (in purple), predictions from a second-generation LASSO model developed by the NEA (in teal) and a *seasonal baseline* model only including weekly random effects as shown in Supplementary Figure 2 (in orange).

Methodology for the second generation NEA LASSO model was as follows. Weekly cases were log-transformed such that the outcome = log(cases +1). Model covariates include population data (i.e., midyear population size for residents and foreigners), weather data (i.e., weekly mean temperature, weekly maximum temperature, number of hours of high temperature, > 27.5°C, weekly relative humidity, and absolute humidity), vector surveillance data (i.e., weekly breeding percentage, defined as the number of *Aedes aegypti* breeding sites found divided by the total number of *Aedes* breeding site found). Model covariates for case, weather and vector surveillance data were lagged with lags from 1 to 20 weeks. The model also included trend and seasonality components from 2000 onwards using breaks the breakfast package (4). We set the minimal number of observations in each segment to 2 years with breaks = 1. In the forecasting phase, the trend and seasonality of the prior year were used as model covariates. The LASSO model was implemented using the *glmnet* package with a tidymodels framework (5,6). Separate models were constructed for each forecast horizon (2, 4, 6, 8 weeks). In each model, the lambda (penalty) was tuned by grid search with 50 levels from 1e-10 to 1, with the best lambda determined by lowest root mean squared error (RMSE). After the initial training (up to end of 2018), the model is retrained every 6 months, following an expanding training data design. During retraining, the trend and seasonality components were recalculated and values from the year prior were used as covariate for forecasting. Forecast confidence intervals were computed from out-of-sample residuals (i.e., using the last 6 months). The lambda parameter was not retuned and the value from the initial training was used throughout.

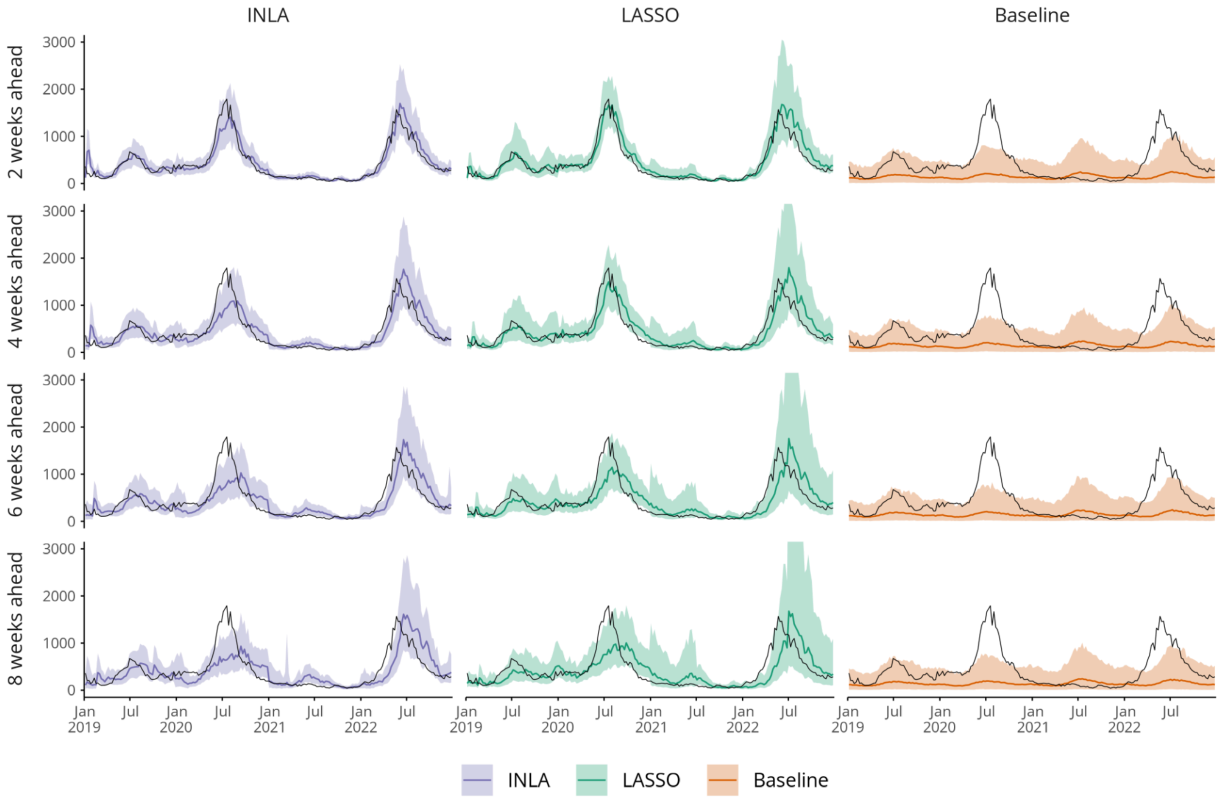

INLA
LASSO
Baseline
2 weeks ahead
4 weeks ahead
6 weeks ahead
8 weeks ahead
0
1000
2000
3000
Jan 2019
Jul
Jan 2020
Jul
Jan 2021
Jul
Jan 2022
Jul
INLA
LASSO
Baseline

Figure 8: Comparing predictive performance of proposed INLA model with the National Environment Agency's LASSO model

Figure showing forecast metrics for each model across all forecast horizons for the 2019, 2020, 2021 and 2022 dengue seasons. From top to bottom these show: weighted interval score, interval coverage %, bias. Interval coverage shows the percentage of observations falling inside a given prediction interval. A perfectly calibrated forecast would have coverage equal to the nominal prediction interval; that is, 95% coverage equal to 95, as indicated by dashed horizontal lines. Bias measures the relative tendency of the model to over- or under-predict, and is bounded between -1 and 1, with 0 indicating unbiased forecasts. The weighted interval score (WIS) is an approximation of the continuous ranked probability score (CRPS) for quantile based forecasts. It can take values between 0 and infinity, with smaller values indicating better performance.

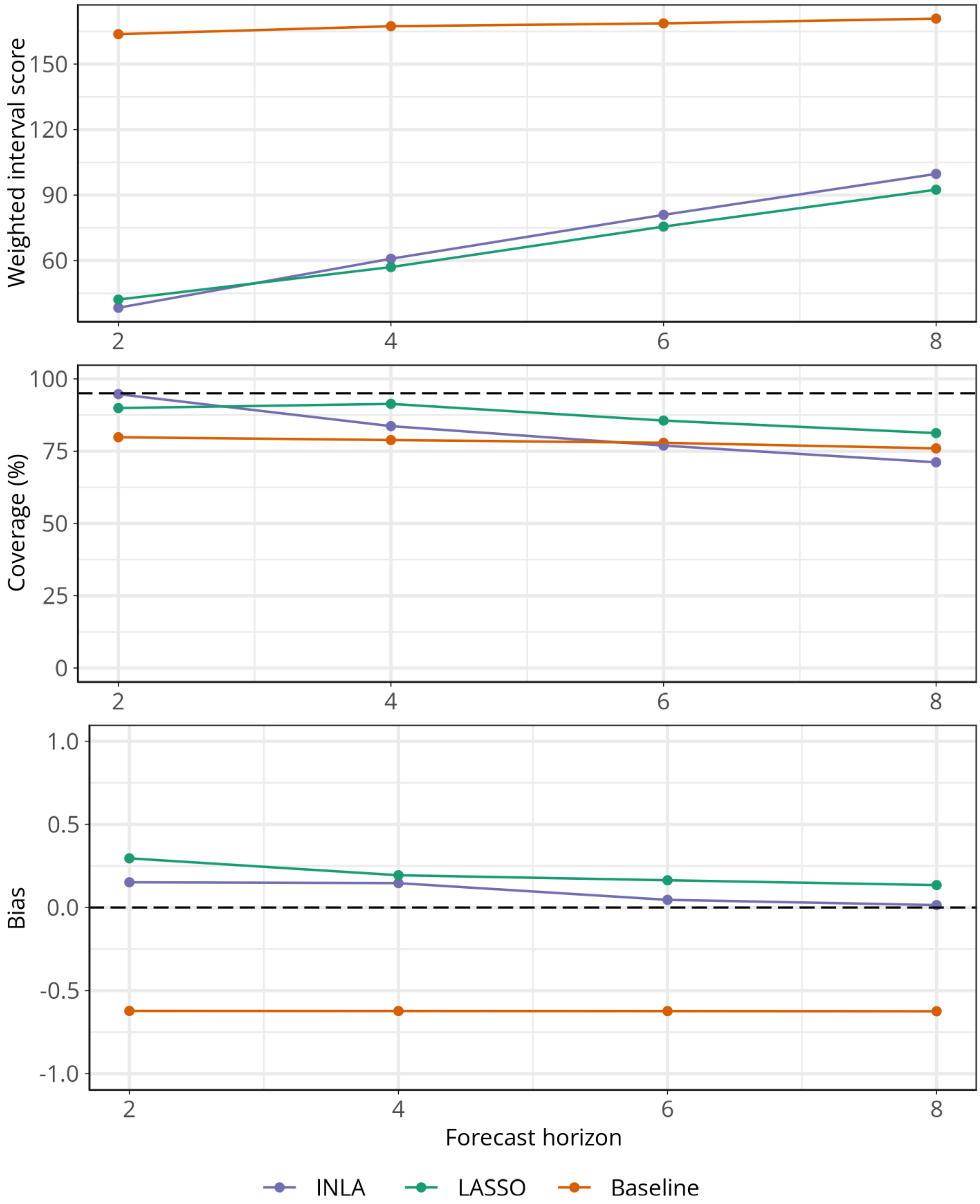

Weighted interval score
60
90
120
150
2
4
6
8
Coverage (%)
0
25
50
75
100
Bias
-1.0
-0.5
0.0
0.5
1.0
Forecast horizon
INLA
LASSO
Baseline

Figure 9: Model fit and predictions for Project *Wolbachia* counterfactual analysis

Figure comparing predicted dengue cases using the *climate and serotype* model with observed cases from 2000 – 2023. The model was tuned using data until 26th June 2022 (indicated with a vertical dashed line) before the start of a randomised controlled trial of *Wolbachia* releases in Singapore. To estimate the impact of *Wolbachia* releases, out-of-sample predictions were generated from this point to the end of 2023 using the *climate and serotype* model. Here, the black line shows the data. The grey line shows model predictions for the model tuning period, representing in-sample model fit, with the associated 95% credible interval shown in the shaded grey area. Out-of-sample model predictions generated for the counterfactual period are shown in purple with the associated 95% prediction interval in the shaded purple area.

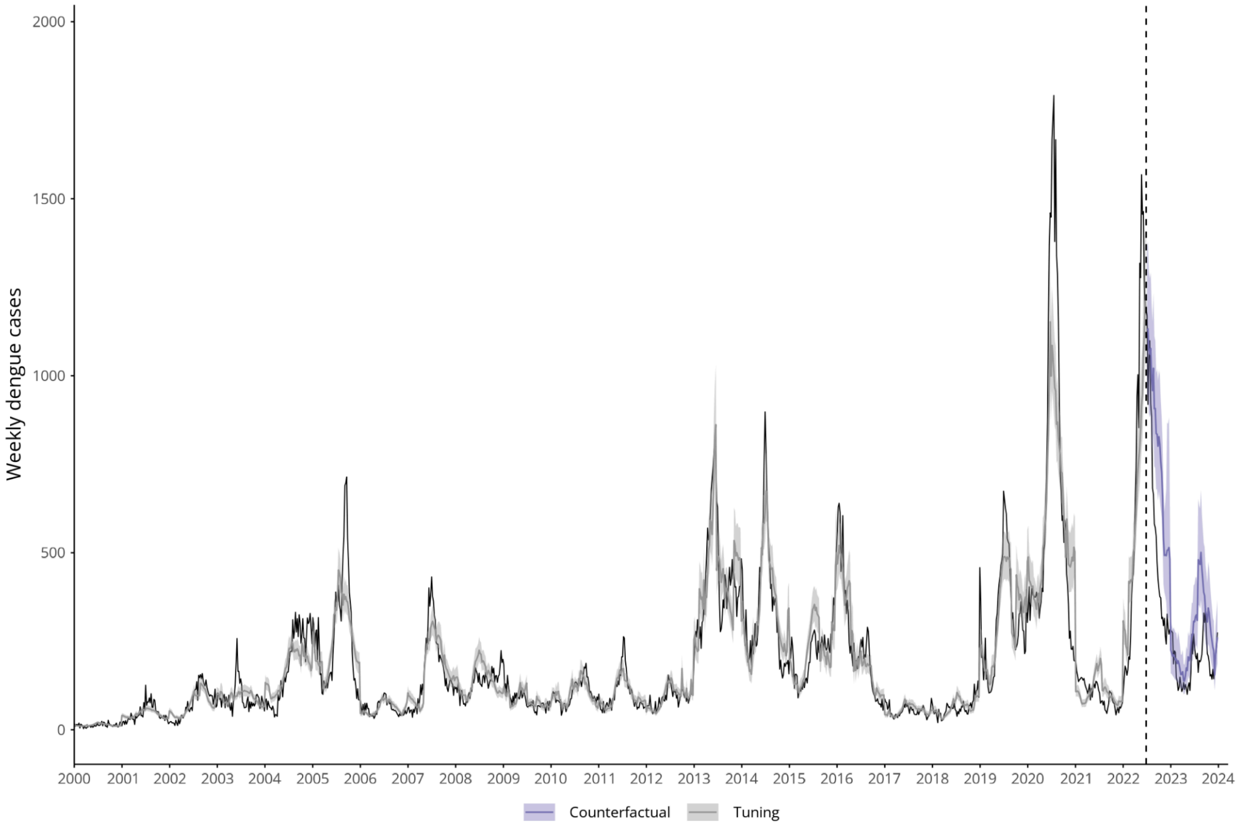

Table 6: Sensitivity analysis - estimated potential impact of Project *Wolbachia* releases with alternate training end dates

In our main analysis we used a training set end date of 26th June 2022, corresponding to the beginning of a multi-site field study involving releases targeting over 150,000 households. This was a significant step-up from the earlier field trials of releases from mid 2018, primarily conducted in four towns (7). We conducted sensitivity analysis to explore the impact of alternate training set end dates on the estimated number of cases averted in the 2023 dengue season. We found that using earlier training set end dates of 10th May 2020 (corresponding to the beginning of larger targeted *Wolbachia* releases) and 1st July 2018 (corresponding to the beginning of small scale releases) led to lower estimated numbers of cases averted (8). This is counterintuitive as we would expect a late training set end date to result in an underestimate of cases averted due to *Wolbachia* releases as some of the effect of releases could have been incorporated into other model covariate estimates, particularly the flexible random effects structure. However, as outbreak sizes have increased year on year, with particularly large outbreaks in 2020 and 2022, the use of earlier training set end dates do not allow the model to learn from these large outbreaks and so result in lower predicted case counts for 2023.

Training set end date	Date reference	Prediction end date	Observed cases	Predicted counterfactual cases (95% CrI)	Cases averted (according to median predicted value)
26/06/2022	Project *Wolbachia* multi-site field study begins	24/12/2023	9959	13748 (9943 - 18659)	3789
10/05/2020	Larger Project *Wolbachia* releases begin	24/12/2023	9959	11218 (8069 - 15537)	1259
01/07/2018	Small scale Project *Wolbachia* releases begin	24/12/2023	9959	10589 (7555 - 15061)	630

References

1. Simpson D, Rue H, Riebler A, Martins TG, Sørbye SH. Penalising Model Component Complexity: A Principled, Practical Approach to Constructing Priors. Stat Sci. 2017 Feb;32(1):1–28.

2. Bosse NI, Abbott S, Cori A, Leeuwen E van, Bracher J, Funk S. Scoring epidemiological forecasts on transformed scales. PLOS Comput Biol. 2023 Aug 29;19(8):e1011393.

3. Shi Y, Liu X, Kok SY, Rajarethinam J, Liang S, Yap G, et al. Three-Month Real-Time Dengue Forecast Models: An Early Warning System for Outbreak Alerts and Policy Decision Support in Singapore. Environ Health Perspect. 2016 Sep;124(9):1369–75.

4. Verbesselt J, Masiliūnas D, Zeileis A, Hyndman R, Appel M, Jung M, et al. bfast: Breaks for Additive Season and Trend [Internet]. 2024 [cited 2025 Jan 13]. Available from: https://cran.r-project.org/web/packages/bfast/index.html

5. Friedman J, Hastie T, Tibshirani R, Narasimhan B, Tay K, Simon N, et al. glmnet: Lasso and Elastic-Net Regularized Generalized Linear Models [Internet]. 2023 [cited 2025 Jan 13]. Available from: https://cran.r-project.org/web/packages/glmnet/index.html

6. Kuhn [aut M, cre, Wickham H, Software P, PBC. tidymodels: Easily Install and Load the 'Tidymodels' Packages [Internet]. 2024 [cited 2025 Jan 13]. Available from: https://cran.r-project.org/web/packages/tidymodels/index.html

7. Lim JT, Bansal S, Chong CS, Dickens B, Ng Y, Deng L, et al. Efficacy of Wolbachia-mediated sterility to reduce the incidence of dengue: a synthetic control study in Singapore. Lancet Microbe. 2024 May 1;5(5):e422–32.

8. Wolbachia-Aedes Mosquito Suppression Strategy [Internet]. [cited 2024 Jun 18]. Available from: https://www.nea.gov.sg
