# Supplementary material for: Climate variation and serotype competition drive dengue outbreak dynamics in Singapore
Source: Nat Commun. 2025 Nov 22;16:11364. doi: 10.1038/s41467-025-66411-6 (PMC12727700; doi:10.1038/s41467-025-66411-6)
Supplement: Supplementary file 3 — Reporting Summary [file 41467_2025_66411_MOESM3_ESM.pdf]

nature portfolio

Corresponding author(s): Emilie Finch and Rachel Lowe

Last updated by author(s): 23/09/24

Reporting Summary

Nature Portfolio wishes to improve the reproducibility of the work that we publish. This form provides structure for consistency and transparency in reporting. For further information on Nature Portfolio policies, see our Editorial Policies and the Editorial Policy Checklist.

Statistics

For all statistical analyses, confirm that the following items are present in the figure legend, table legend, main text, or Methods section.

n/a	Confirmed	
☐	☒	The exact sample size (*n*) for each experimental group/condition, given as a discrete number and unit of measurement
☐	☒	A statement on whether measurements were taken from distinct samples or whether the same sample was measured repeatedly
☒	☐	The statistical test(s) used AND whether they are one- or two-sided<br>*Only common tests should be described solely by name; describe more complex techniques in the Methods section.*
☐	☒	A description of all covariates tested
☐	☒	A description of any assumptions or corrections, such as tests of normality and adjustment for multiple comparisons
☐	☒	A full description of the statistical parameters including central tendency (e.g. means) or other basic estimates (e.g. regression coefficient) AND variation (e.g. standard deviation) or associated estimates of uncertainty (e.g. confidence intervals)
☒	☐	For null hypothesis testing, the test statistic (e.g. *F*, *t*, *r*) with confidence intervals, effect sizes, degrees of freedom and *P* value noted<br>*Give P values as exact values whenever suitable.*
☐	☒	For Bayesian analysis, information on the choice of priors and Markov chain Monte Carlo settings
☒	☐	For hierarchical and complex designs, identification of the appropriate level for tests and full reporting of outcomes
☐	☒	Estimates of effect sizes (e.g. Cohen's *d*, Pearson's *r*), indicating how they were calculated

Our web collection on statistics for biologists contains articles on many of the points above.

Software and code

Policy information about availability of computer code

Data collection	No software was used to collect the data in this study.
Data analysis	All code used for this analysis are available at: https://github.com/EmilieFinch/dengue-singapore. This analysis was performed using R 4.1.1 and INLA_21.11.22. Other R packages used in this analysis include: scoringutils 1.2.1 and pROC 1.18.4.

For manuscripts utilizing custom algorithms or software that are central to the research but not yet described in published literature, software must be made available to editors and reviewers. We strongly encourage code deposition in a community repository (e.g. GitHub). See the Nature Portfolio guidelines for submitting code & software for further information.

Data

Policy information about availability of data

All manuscripts must include a data availability statement. This statement should provide the following information, where applicable:

- Accession codes, unique identifiers, or web links for publicly available datasets
- A description of any restrictions on data availability
- For clinical datasets or third party data, please ensure that the statement adheres to our policy

Data used for this study are available at: https://github.com/EmilieFinch/dengue-singapore. Data on dengue serotype frequencies shown in Figure 1 are not included in the Github repository as they are not publicly available. However, these data are not needed to reproduce the analysis.

Research involving human participants, their data, or biological material

Policy information about studies with human participants or human data. See also policy information about sex, gender (identity/presentation), and sexual orientation and race, ethnicity and racism.

Reporting on sex and gender	In this study we use weekly epidemiological case data. As we did not have access to individual level data or cases broken down by sex or gender we could not conduct sex and gender-based analyses.
Reporting on race, ethnicity, or other socially relevant groupings	In this study we use weekly epidemiological case data. We did not have access to individual level data or cases broken down by race, ethnicity or other socially relevant groupings and we could not conduct analyses at this level. As we were conducting a time-series analysis, confounding by these factors is only likely if they are time-varying. To account for this and other unmeasured potential confounders (such as vector control measures) we include a yearly random effect within the model framework.
Population characteristics	In this study we use aggregated weekly case data and do not have access to individual-level data on population characteristics.
Recruitment	Our study includes all reported dengue cases from 1 January 2000 - 31 December 2022. Dengue case reporting by clinicians and laboratories is mandatory in Singapore, the study setting. We also used data on serotype frequencies from the Environmental Health Institute's viral surveillance program, which serotypes a subset of dengue cases each week.
Ethics oversight	This study was exempted from formal bioethics review by both LSHTM and the national Singaporean ethics procedure as it is not considered human biological research. The data used was collected as part of Singapore's national dengue surveillance programme. Data on dengue cases and serotype was approved by the Ministry of Health, Singapore for use in this study.

Note that full information on the approval of the study protocol must also be provided in the manuscript.

Field-specific reporting

Please select the one below that is the best fit for your research. If you are not sure, read the appropriate sections before making your selection.

☒ Life sciences ☐ Behavioural & social sciences ☐ Ecological, evolutionary & environmental sciences

For a reference copy of the document with all sections, see nature.com/documents/nr-reporting-summary-flat.pdf

Life sciences study design

All studies must disclose on these points even when the disclosure is negative.

Sample size	234,358 cases in 1200 weeks of data.
Data exclusions	Weekly dengue case data between 1 Jan 2000 and 31 Dec 2022 were analysed. There was no missing or excluded data.
Replication	We checked for reproducibility of study findings during model verification and evaluation. We also conducted internal code review, checking that the code could be run independently and the results reproduced.
Randomization	This is an observational study and as such we did not allocate experimental groups.
Blinding	This study did not include experimental group and as such there was no blinding for investigators.

Reporting for specific materials, systems and methods

We require information from authors about some types of materials, experimental systems and methods used in many studies. Here, indicate whether each material, system or method listed is relevant to your study. If you are not sure if a list item applies to your research, read the appropriate section before selecting a response.

Materials & experimental systems

n/a	Involved in the study	
☒	☐	Antibodies
☒	☐	Eukaryotic cell lines
☒	☐	Palaeontology and archaeology
☒	☐	Animals and other organisms
☒	☐	Clinical data
☒	☐	Dual use research of concern
☒	☐	Plants

Methods

n/a	Involved in the study	
☒	☐	ChIP-seq
☒	☐	Flow cytometry
☒	☐	MRI-based neuroimaging

Plants

Seed stocks	*Report on the source of all seed stocks or other plant material used. If applicable, state the seed stock centre and catalogue number. If plant specimens were collected from the field, describe the collection location, date and sampling procedures.*
Novel plant genotypes	*Describe the methods by which all novel plant genotypes were produced. This includes those generated by transgenic approaches, gene editing, chemical/radiation-based mutagenesis and hybridization. For transgenic lines, describe the transformation method, the number of independent lines analyzed and the generation upon which experiments were performed. For gene-edited lines, describe the editor used, the endogenous sequence targeted for editing, the targeting guide RNA sequence (if applicable) and how the editor was applied.*
Authentication	*Describe any authentication procedures for each seed stock used or novel genotype generated. Describe any experiments used to assess the effect of a mutation and, where applicable, how potential secondary effects (e.g. second site T-DNA insertions, mosiacism, off-target gene editing) were examined.*
